# Acute emotional stress as a trigger for intraocular pressure elevation in Glaucoma

**DOI:** 10.1186/s12886-019-1075-4

**Published:** 2019-03-08

**Authors:** Kevin Gillmann, Kirsten Hoskens, Kaweh Mansouri

**Affiliations:** 1 Glaucoma Research Center, Montchoisi Clinic, Swiss Visio Network, Lausanne, Switzerland; 2 Department of Ophthalmology, University of Colorado School of Medicine, Denver, CO USA

**Keywords:** Glaucoma, Intraocular pressure, Stress, Anxiety, Mental health, Risk factor

## Abstract

**Background:**

Stress-induced activation of the sympathetic nervous system leads to a cascade of metabolic reactions. Emotional stress is a more specific form of stress in which the stressor is a psychological response to a situation subjectively perceived as traumatic. Stress hormones can have a wide range of effects on the body, however, it is still unclear if and how it can affect ophthalmic physiology. This report presents a case of severe ocular hypertension in which emotional stress was the only cause elicited, and explores potential aggravating factors.

**Case presentation:**

A 78-year-old, personality type A, lady with a history of pseudo-exfoliative glaucoma presented with an acute asymmetrical raise in intraocular pressure (IOP) immediately following a family breakdown. Her IOP had previously remained stable following a deep sclerectomy in the right eye and an Ex-PRESS shunt in the left eye. Her examination was entirely normal otherwise, with a patent filtration and diffuse bleb as confirmed with anterior segment OCT imaging. Near-normalisation of her IOP was observed within 24 h, concomitantly with the reduction of her stress levels. No other cause for the transient acute hypertensive episode were found.

**Conclusions:**

This case report suggests that acute emotional stress could severely affect IOP in patients suffering from glaucoma. This could be important when looking after glaucoma patients. It would also suggest that the personnality types, and the emotional and social context are more factors to take into account in glaucoma studies. These observations are based on a single case report and would need to be verified on a larger scale.

## Background

The exact pathophysiology and biological mechanisms underlying glaucomatous changes are still not completely understood [1]. It has been clearly documented, however, that intraocular pressure (IOP) plays a critical role in the disease processes and that elevated IOP is one of the main risk factors for glaucoma progression [2, 3]. Conversely, obtaining low IOPs in glaucomatous eyes was shown to slow disease progression and improve long-term visual prognosis [4–7].

Stress is medically defined as a disruption in the homeostatic state of an organism. On a multi-organ scale it has long been known that hormones, and more specifically stress hormones, can have a wide range of effects on the body in the human and the animal model alike [8–11]. Stress-induced activation of the sympathetic nervous system leads to a cascade of metabolic reactions known as stress response. This response, primarily mediated through the hypothalamic-pituitry-adrenal axis, interferes with the physiological levels of circulating hormones, including cortisol [12]. Variation in these hormones levels can result in systemic diseases such as high blood-presure or atherosclerosis [13]. Emotional stress is a more specific form of stress in which the stressor is a psychological response to a situation subjectively perceived as traumatic. Its subjective nature and the ethical questions associated with the infliction of severe emotional distress to human subjects makes this form of stress more difficult to study in a controlled setting. However, there are extensive reports of how emotionally stressful situations can affect cardiovascular homeostasis [14] and be responsible for acute pathological changes, such as in Takotsubo syndrome [15]. Despite of these evidence, it is still unclear if and how these matabolic changes can affect ophthalmic physiology. Several small studies suggest a mild but statistically significant IOP-increasing effect of stress [16] and, conversely, anecdotal data can be found in the litterature, suggesting that relaxation may have the opposite effect [17].

We report a case in which acute emotional stress was identified as the only trigger for severe IOP elevation and discuss the implications of this case for future research and glaucoma management in the context of our current knowledge.

## Case presentation

A 78-year-old lady of Hispanic ethnicity, with pseudo-exfoliative glaucoma (PEXG) and exudative age-related macular degeneration (AMD) presented for a routine follow-up appointment in a large tertiary ophthalmology clinic with an acutely raised IOP in the left eye. Her past surgical history comprised of combined Ex-PRESS glaucoma shunt and cataract surgery in the left eye 12 years previously. Her right eye underwent combined deep sclerectomy and cataract surgery 11 years prior to the present presentation, with a subsequent YAG goniopuncture performed 2 years post-operatively. Her IOP had since remained relatively stable between 14 and 19 mmHg in the right eye, and 16–21 mmHg in the left eye with a topical therapy of latanoprost (Xalatan, Pfizer PFE Switzerland GmbH, Switzerland) and timolol 0.1% (Timogel, Théa Pharma SA, Switzerland). The AMD had remained stable since completing a series of three ranibizumab (Lucentis, Novartis Pharma, Switzerland) intravitreal injections in the left eye 4 months previously.

At the described presentation, the patient was completely asymptomatic with no reported pain or discomfort and a best corrected visual acuity of 10/10 in the right eye and 7/10 in the left eye. On examination, her IOP was 18 mmHg in the right eye and 48 mmHg in the left eye, as measured with a Golmann tonometer, with a pachymetry of 552 and 555 μm in the right and the left eye respectively.

**Fig. 1:**
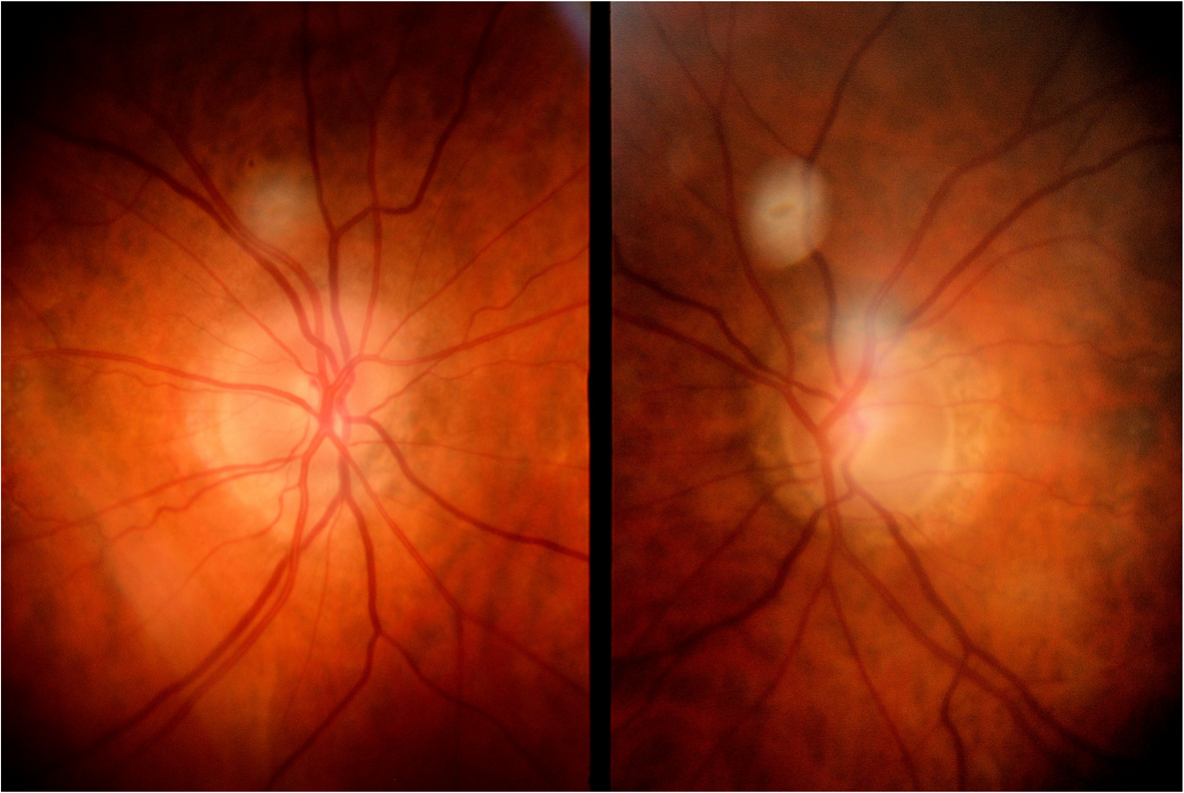
Images of the optic discs showing asymmetrical cupping. The left optic nerve head (right image) has a cup/disc ratio of 0.7

**Fig. 2:**
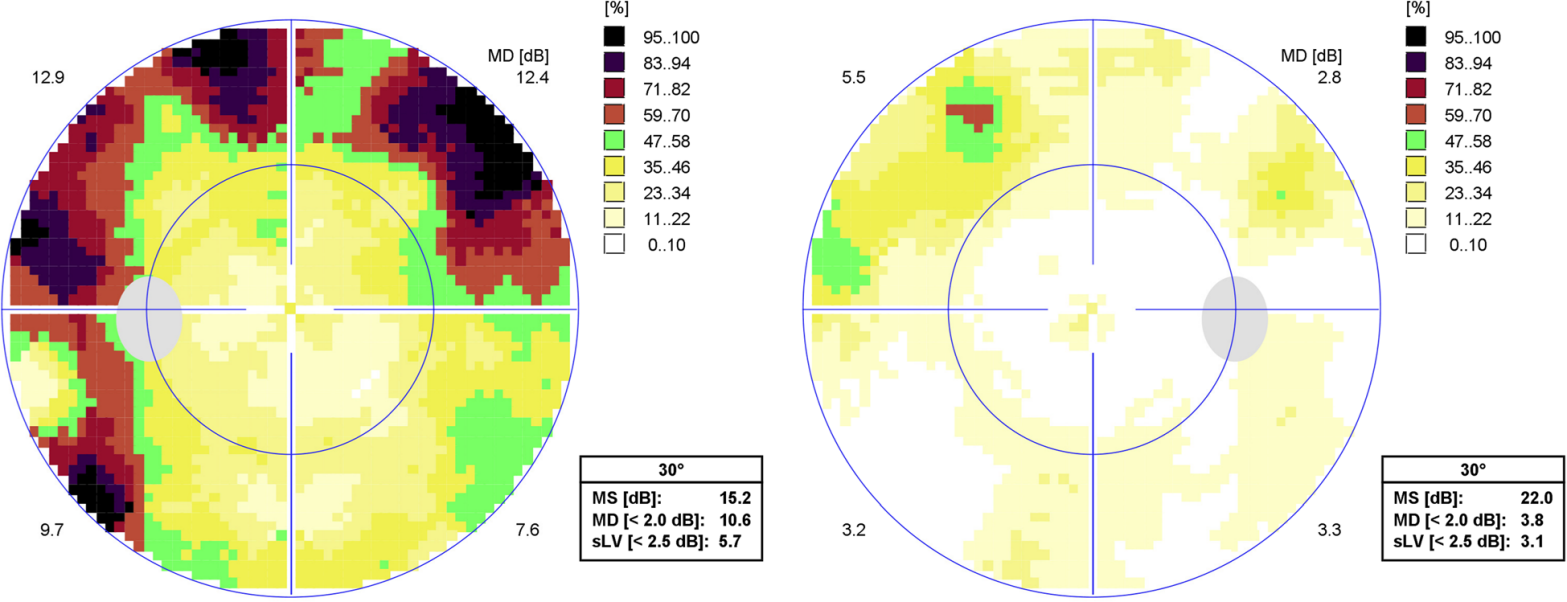
30° Octopus visual field examination showing marked superior and inferior arciform scotomata in the left eye (left image) with a mean deviation of 10.6 dB, and a mild superior arciform scotoma in the right eye (right image) with a mean deviation of 3.8 dB. (MS: Mean sensitivity; sLV: Square root of lost variance)

**Fig. 3:**
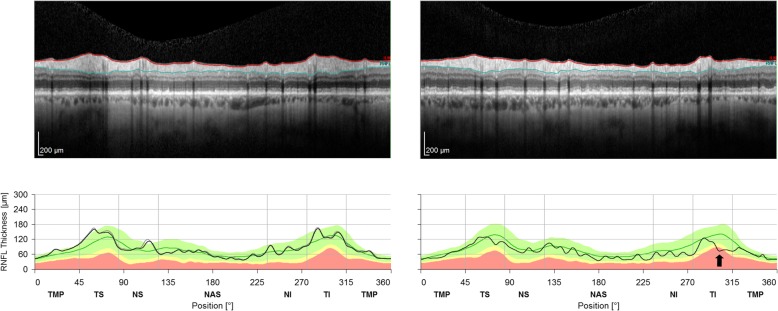
OCT retinal nerve fibre layer (RNFL) analysis confirming mild generalised thinning of RNFLs, in the presence of a focal inferotemporal deficit (black arrow) in the left eye (right image)

Slit-lamp examination confirmed quiet anterior chambers with intraocular lenses in place, and good-sized diffuse filtration blebs in both eyes, with two scleral sutures in situ within the left bleb. Gonioscopic examination was unremarkable showing open angles in both eyes, with an open trabeculo-Descemetic membrane in the right eye and an Ex-PRESS shunt in situ in the left eye, the position and patency of which was confirmed with an anterior segment optical coherence tomography (OCT) (Spectralis OCT, Heidelberg Engineering AG, Germany). The latter also confirmed the functional and diffuse appearances of both filtration blebs. Fundus examination, visual fields and OCT imaging were stable, with extensive drusens in both eyes, no recurrence of macula oedema, normal retinal vasculature, and a cup/disc ratio of 0.6 in the right eye and 0.7 in the left, on 1.4 mm-diameter papillae (Figs. 1, 2, 3).

Subjective history confirmed good adherence to the medical therapy and the absence of any recent physically traumatic incident. However, the patient volunteered going through a period of severe emotional stress due to a recent family breakdown and an emotional argument immediately prior to the appointment. Her personality was subjectively assessed as type A by the medical team.

She was commenced on a combination of topical timolol and dorzolamide (Cosopt, Santen, Japan) and brimonidine (Alphagan, Allergan, Dublin, Ireland) twice a day in the left eye, and a daily dose of 500 mg acetazolamide (Diamox, Vifor Pharma, Switzerland) administered orally, in an attempt to promptly normalise IOP and preserve nerve fibres.

The next day, IOP had normalised to 10 mmHg and 16 mmHg in the right and the left eye respectively. The systemic medications were reduced and stopped, and the IOP returned to near-baseline levels, with subsequent measures between 12 and 16 mmHg in the right eye and 18–23 mmHg in the left eye, as shown in Fig. 1. In the meantime, the patient reported some subjective reduction in her levels of stress despite ongoing anxiety and a difficult familial situation.

**Fig. 4:**
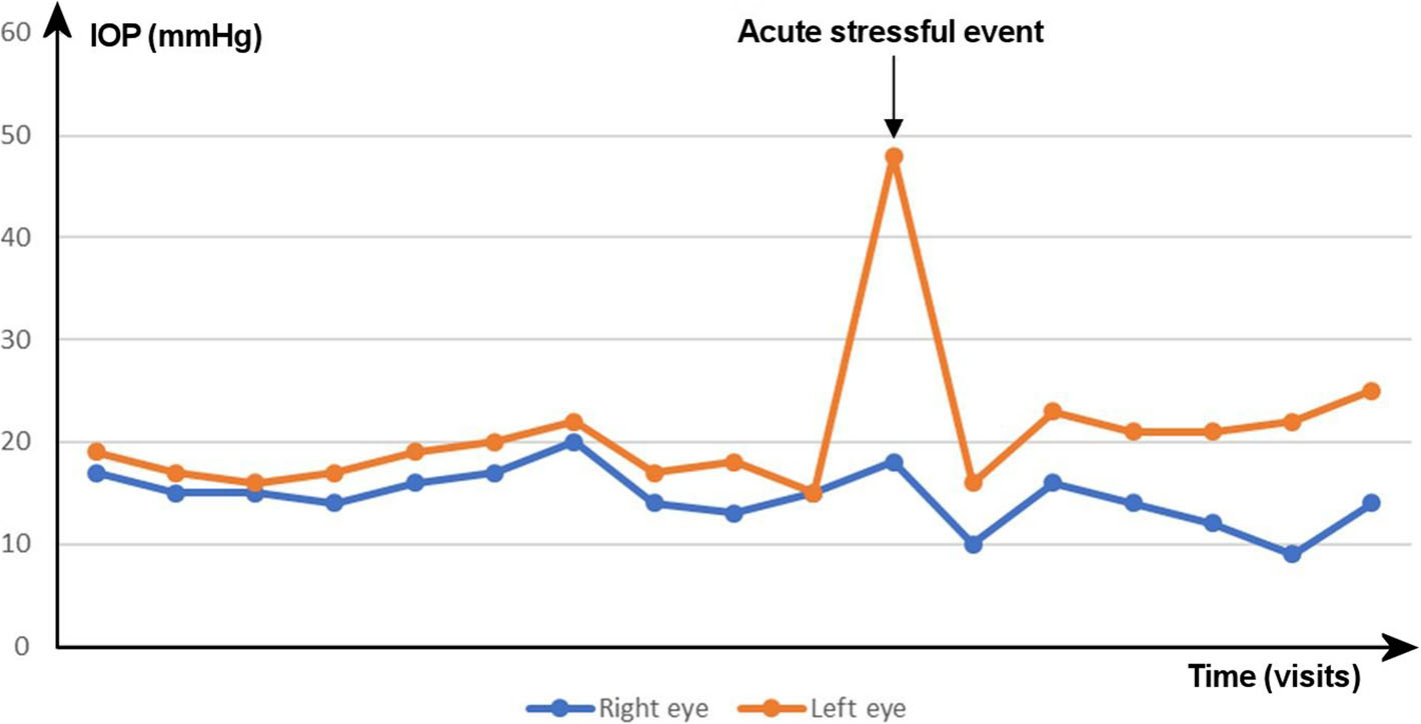
Long-term progression of IOP in the right and the left eye, before and after the acute stressful event

**Fig. 5:**
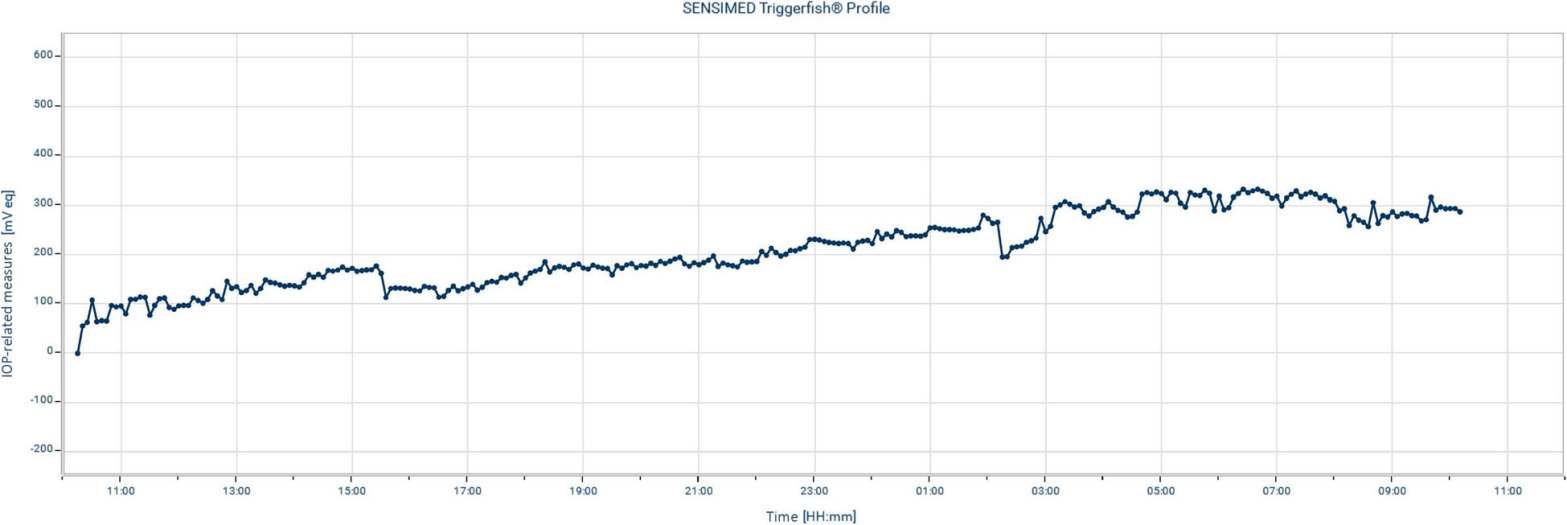
24-h trace of IOP-related variations in the left eye recorded with a contact lens sensor. Triggerfish sensors measure fluctuations in corneal shape (in mV) that tend to reflect IOP variations. The recorded curve above shows relative stability over the entire duration of the recording

Two months later, the IOP in both eyes was still stable under topical timolol and dorzolamide in the left eye (Fig. 4). To exclude inter-measures and diurnal variations, twenty-four-hour monitoring of IOP-related variations using a Triggerfish contact lens sensor (Sensimed SA, Lausanne, Switzerland) was performed. It suggested relative stability of the pressures through the day and at night, with minimal changes following the instillation of topical therapy (Fig. 5). Posterior needling combined with suture lysis was performed to further improve IOP control in the left eye, however despite mild initial improvement, IOPs stabilised at 22 mmHg after 2 months. A XEN-augmented Baerveldt procedure was carried out, achieving an unmedicated IOP of 16 mmHg at 1 month [18].

## Discussions and conclusions

This case report suggests that severe emotional stress could acutely affect IOP control in patients suffering from PEXG. This is primarily supported by the absence of identified anatomical or physiological outflow obstruction, in the presence of large functional filtration blebs. Nonetheless, PEXG is known for its instability and aggressive nature, and the persistent decompensation perpetuated by unidentified factors indicates probable mixed mechanisms. The relative stability of IOPs prior to the stressful trigger associated with the near-resolution of the ocular hypertensive crisis as the emotional situation of the patient improved, however, are further indicators that emotional stress at least contributed to the acute crisis.

In this case, the response is clearly asymmetrical, with a significantly greater increase in IOP in the left eye compared to the right. Despite several descriptions of IOP discrepancies between the eyes of patients undergoing bilateral glaucoma surgery in the literature [19, 20], this observation was rather unexpected. It could, however, be accounted for by several differences between the two eyes. First, by the structural differences following surgery. The right eye underwent non-penetrating deep sclerectomy with subsequent Nd:YAG laser goniopuncture, creating a direct connection between the anterior chamber and the subconjunctival space, while the left eye underwent Ex-PRESS shunt implantation. The technique used in the latter is similar to those of penetrating trabeculectomy, only after the scleral flap is raised, a metal shunt instead of a scleral punch is used to gain access to the anterior chamber [21]. Despite providing more intrinsic resistance than trabeculectomy, the Ex-PRESS shunt only delivers minimal outflow resistance under physiological conditions (0.09 mmHg resistance) and thus similarly relies on the suture of the scleral flap to prevent immediate post-operative hypotony [22]. The tension in the scleral flap creates more outflow resistance than is achieved by the open filtration model of a post-goniopuncture deep sclerectomy, which could account for the discrepencies between the two eyes. Stress-related elevation of IOP in an eye that underwent filtration surgery, thus bypassing the episclreal venous system, suggests that stress not only has an effect on outflow resistance, but also on the rate of acqueous production, as this has already been observed in an animal model by Niederer et al. [23] Secondly, recent unilateral exposure to ranibizumab could potentially contribute to the observed difference in IOP between the two eyes. In a recent study, Foss et al. [24] has shown that anti-VEGF treatments induced a durable and cumulative increase in IOP after each intravitreal injection, leading to a gradually widening discrepancy in IOP between the treated and the untreated eye. However, despite being significant, the described effect was relatively small. Thus we do not expect it to be solely responsible for the difference observed in this case. Nevertheless, other studies have shown that anti-VEGF agents were associated with a decrease in prostaglandin-I 2 (PGI2) production [25], a type of prostaglandin that was shown to suppress some of the effects of cortisol in the animal model [26]. It could therefore be theorised that anti-VEGF therapies, through lower levels of PGI2, might lead to an unopposed action of cortisol, thus locally potentiating its effect and potentially increasing the effect of stress. Finally, the severity of the disease might be another factor responsible for the difference in IOP between the two eyes. In their study Gottanka et al. showed that, in eyes with PEXG, maximum IOP was directly correlated both to the amount of axonal damage observed at the optic nerve and to the amount of pseudo-exfoliative (PEX) material within Schlemm’s canal [27]. In the present case, higher IOP were noted in the left eye, where PEXG was clinically more severe. Therefore, it could be suspected that this would be associated with more PEX material within Schlemm’s canal, which might be another factor responsible for higher IOP in this eye.

Several studies have suggested that hormonal variations and emotional responses could affect IOP in human models, as illustrated by Brody et al. who observed a 1.3 mmHg increase in IOP after the subjects were asked to partake in complex mental arithmetic tasks [28], or by Mansouri et al. who described a substantial reduction in IOP associated with sexual activity and orgasm [29]. The impact of emotional stresss on IOP, however, is less documented and studies observations are contradictory.

In 2018, Méndez-Ulrich et al. found that IOP was on average 2.3 mmHg higher in a group of self-reported nervous volunteers compared to the group of low-anxiety subjects [30]. The former group was also found to have a higher heart-rate than the latter, but no significant difference in blood pressure was found. A previous study by Ismail et al. supported this finding when they noted that melatonin-mediated anxiety reduction prior to cataract surgery was associated with a similar diminution in IOP, but reported a statistically significant diminution in mean arterial blood pressure with no change to the heart-rate [31]. In this study, IOP was measured on several occasions including 90 min prior to surgery (before pre-medication was given), upon entering the operating theatre and post-operatively. It is interesting to note that, in the unmedicated control group, none of these IOP measures showed any statistically significant variation, while one might expect different levels of anxiety upon entering the operating theatre or after the surgery is complete. In a comparable study on the effect of melatonin on stress and IOP, Khezri et al. contradicts these results when they described that pharmacological reduction in anxiety minimized its cardiovascular impact but had no significant effect on IOP [32].

These variations in observations of the effect of anxiety can be accounted for by several factors. Firstly, as Terracciano et al. highlighted in their study analysing the incidence of white coat syndrome in different personality types, in which they observed that anxious personality types tended to find the clinical context of a health-check reassuring, emotional stress is a subjective experience and it can be difficult to theorise on what will be considered a stressful experience for a majority of subjects [33]. Secondly, it was shown that subjects’ personnality types and ages can have a significant impact on IOP variations, with type A personalities showing the greatest variability [34]. These characteristics were not analysed or corrected for in most of the studies reviewed. Lastly, it is known that patients suffering from glaucoma, and even more so PEXG, present wider IOP variations during the day [35]. The reviewed studies on melatonin did not specifically include or exclude glaucomatous eyes, the proportion of which could potentially affect the results.

In this context, our case suggests that acute emotional stress could potentially trigger or precipitate a severe ocular response in the form of a marked IOP rise. The extent of the response to the stressful stimuli could be influenced by several factors including a diagnosis of glaucoma, the type of glaucoma, the surgical history of the eye, the personnality type of the patient and the percieved severity of the emotional stress. This could be important to keep in mind when looking after glaucoma patients, especially in younger populations in which a diagnosis of glaucoma tends to be associated with higher rates of anxiety [36]. It would also suggest that the personnality types, and the emotional and social context are more factors to take into account in glaucoma studies. These observations and hypotheses are based on a single, incidental case report and would need to be verified on a larger scale, with a structured and standardised approach.

## Abbreviations

AMD: age-related macular degeneration
IOP: intraocular pressure
Nd:YAG: neodymium-doped yttrium aluminum garnet
OCT: optical coherence tomography
PEX: pseudo-exfoliative
PEXG: pseudo-exfoliative glaucoma
VEGF: vascular endothelial growth factor
